# Characteristics and impact of interventions to support healthcare providers’ compliance with guideline recommendations for breast cancer: a systematic literature review

**DOI:** 10.1186/s13012-023-01267-2

**Published:** 2023-05-22

**Authors:** Ignacio Ricci-Cabello, Darla Carvallo-Castañeda, Adrián Vásquez-Mejía, Pablo Alonso-Coello, Zuleika Saz-Parkinson, Elena Parmelli, Gian Paolo Morgano, David Rigau, Ivan Solà, Luciana Neamtiu, Ena Niño-de-Guzmán

**Affiliations:** 1 Balearic Islands Health Research Institute (IdISBa), Palma, Spain; 2 Primary Care Research Unit of Mallorca, Balearic Islands Health Service, Palma, Spain; 3 CIBER de Epidemiología y Salud Pública (CIBERESP), Madrid, Spain; 4 Facultad de Medicina Humana, Universidad Nacional Mayor de San Marcos, Lima, Peru; 5 Iberoamerican Cochrane Centre-Department of Clinical Epidemiology and Public Health, Biomedical Research Institute Sant Pau (IIB Sant Pau), Barcelona, Spain; 6 European Commission, Joint Research Centre (JRC), Ispra, Italy; 7 Cancer Prevention and Control Programme, Catalan Institute of Oncology, IDIBELL, Hospitalet de Llobregat, Barcelona, Spain

**Keywords:** Breast cancer, Clinical guidelines, Compliance, Interventions, Systematic literature review

## Abstract

**Background:**

Breast cancer clinical practice guidelines (CPGs) offer evidence-based recommendations to improve quality of healthcare for patients. Suboptimal compliance with breast cancer guideline recommendations remains frequent, and has been associated with a decreased survival. The aim of this systematic review was to characterize and determine the impact of available interventions to support healthcare providers’ compliance with CPGs recommendations in breast cancer healthcare.

**Methods:**

We searched for systematic reviews and primary studies in PubMed and Embase (from inception to May 2021). We included experimental and observational studies reporting on the use of interventions to support compliance with breast cancer CPGs. Eligibility assessment, data extraction and critical appraisal was conducted by one reviewer, and cross-checked by a second reviewer. Using the same approach, we synthesized the characteristics and the effects of the interventions by type of intervention (according to the EPOC taxonomy), and applied the GRADE framework to assess the certainty of evidence.

**Results:**

We identified 35 primary studies reporting on 24 different interventions. Most frequently described interventions consisted in computerized decision support systems (12 studies); educational interventions (seven), audit and feedback (two), and multifaceted interventions (nine). There is low quality evidence that educational interventions targeted to healthcare professionals may improve compliance with recommendations concerning breast cancer screening, diagnosis and treatment. There is moderate quality evidence that reminder systems for healthcare professionals improve compliance with recommendations concerning breast cancer screening. There is low quality evidence that multifaceted interventions may improve compliance with recommendations concerning breast cancer screening. The effectiveness of the remaining types of interventions identified have not been evaluated with appropriate study designs for such purpose. There is very limited data on the costs of implementing these interventions.

**Conclusions:**

Different types of interventions to support compliance with breast cancer CPGs recommendations are available, and most of them show positive effects. More robust trials are needed to strengthen the available evidence base concerning their efficacy. Gathering data on the costs of implementing the proposed interventions is needed to inform decisions about their widespread implementation.

**Trial registration:**

CRD42018092884 (PROSPERO)

**Supplementary Information:**

The online version contains supplementary material available at 10.1186/s13012-023-01267-2.

Contributions to the literature

- Research has shown that compliance with breast cancer clinical practice guidelines remains suboptimal, leading to increased mortality rates.
- Our study is the first systematic review evaluating interventions to support compliance with breast cancer clinical practice guidelines recommendations, and builds upon previous reviews of this topic in more general contexts. We found that a number of different types of interventions have been developed and evaluated, most of them showing beneficial effects.
- The quality of the evidence is low for provider educational interventions, moderate for provider reminders, and low for multifaceted interventions. For the rest of the interventions identified, the evidence is uncertain.
- This review contributes to recognized gaps in the literature, including ascertaining which types of interventions work best to promote compliance with breast cancer CPGs, as well as identifying new areas for future research.
- Findings from this review may help those practitioners and health decision makers interested in improving the quality and safety of breast cancer healthcare provision by enhancing the uptake of clinical practice guidelines.

## Introduction

Breast cancer is the most common cancer in women with 2.3 million new cases estimated in 2020, accounting for 11.7% of all cancers [1]. It is the fifth leading cause of cancer mortality worldwide, with 685,000 deaths [1]. Breast cancer diagnosis is more frequent in developed countries [2]. Controlling and preventing breast cancer is an important priority for health policy makers [3].

Treatment procedures have rapidly evolved over recent years. As new and precise diagnosis strategies emerged, early treatment and prognosis of breast cancer patients have shown great progresses [4]. Advances in breast cancer screening and treatment have reduced the mortality of breast cancer across the age spectrum in the past decade [5–7]. Although the use of research evidence can improve professional practice and patient-important outcomes, considering also the huge volume of research evidence available, its translation into daily care routines is generally poor [8, 9]. It is estimated that it takes an average of 17 years for only 14% of new scientific discoveries to enter day-to-day clinical practice [10].

Clinical Practice Guidelines (CPGs) provide recommendations for delivering high quality healthcare [11, 12]. However, the impact of CPGs depends not only on their quality, but also on the way and the extent to which they are used by clinicians in routine clinical practice. Large overviews show that approximately 50% of patients receive from general medical practitioners treatments which differ from recommended best practice [13–16]. In the area of breast cancer, previous systematic reviews have shown that compliance with breast cancer CPGs [17], as well as for other types of cancer [18–20], remains suboptimal. A recent systematic review from our research group [21] found large variations in providers´ compliance with breast cancer CPGs, with adherence rates ranging from 0 to 84.3%. Sustainable use of CPGs is also notably poor: after 1 year of their implementation, adherence decreases in approximately half of the cases [22].

Suboptimal compliance with CPGs recommendations could increase healthcare costs if healthcare resources are overused (e.g., overtreatment, overuse of diagnosis or of screening techniques); but also, if they are underused (i.e., increased costs to cover the additional health care needs that people may face with worsening conditions due to under-used resources). Available evidence suggests that outcomes may improve for patients, healthcare professionals and healthcare organizations if decision-makers adhere to evidence-based CPGs [23, 24]. This is supported by a recent meta-analysis from our group [25], which suggests that compliance with CPGs is probably associated with an increase in both, disease-free survival (hazard ratio (HR) = 0.35 (95% CI from 0.15 to 0.82)) and overall survival (HR = 0.67 (95% CI 0.59 to 0.76). Developing interventions to support clinician uptake of breast cancer CPGs is therefore essential for improving healthcare quality and patient important outcomes. Although several interventions to support compliance with breast cancer CPGs have been proposed, no previous study has systematically examined their characteristics and effects.

The aim of this systematic review is to characterize and evaluate the impact of available interventions to support healthcare providers’ compliance with CPGs in breast cancer care.

## Methods

### Design

We conducted a systematic literature review adhering to the PRISMA reporting guidelines [26] (PRISMA 2020 Checklist available at Additional file 1). In this review, we addressed the following two questions: (1) What type of interventions have been used to support healthcare professionals´ compliance with breast cancer CPGs? and; (2) What type of interventions can effectively support healthcare professionals’ compliance with breast cancer CPGs? We registered the protocol in the international prospective register of systematic reviews (PROSPERO registration number CRD42018092884).

### Searches

We searched for systematic reviews and original studies in MEDLINE (through PubMed) and Embase (through Ovid) using predefined search strategies from inception to May 2021 designed and implemented by an information specialist (IS) from the Iberoamerican Cochrane Centre (IS). The search strategies (available in Additional file 2) combined MeSH terms and keywords.

### Study selection

We applied the following inclusion criteria:

- Population: healthcare professionals providing health services related to the prevention or management of breast cancer. All types of healthcare professionals, and from any setting were included.
- Intervention: interventions explicitly aimed at supporting or promoting healthcare professionals’ compliance with available breast cancer CPGs. Such guidelines may address any specific aspect of breast cancer care, including screening, diagnosis, treatment, surveillance or rehabilitation.
- Comparator: any comparator, including also studies not using a comparator group.
- Outcome: quality of breast cancer care (based on healthcare professionals’ compliance rate with breast cancer CPGs recommendations, but also on their knowledge, attitudes or self-efficacy concerning such recommendations); intervention implementation (fidelity, reach, implementation costs), and; patient health-related outcomes (e.g., survival).

We included experimental (randomized controlled and non-randomized controlled trials), observational (before-after, cohort, case-control, cross-sectional, and case studies), and qualitative or mixed-methods studies. Due to constrained resources, we only included studies published in English. One author (of IRC, DC, APVM) screened the search results based on title and abstract. A second author (ENG, LN, ZSP, EP, DC, APVM, GPM) independently reviewed 20% of all references. Two authors independently assessed eligibility based on the full text of the relevant articles. Disagreements were discussed (involving a third author when needed) until consensus was reached.

### Data extraction

One author (ENG, IRC LN, ZSP, EP, DC, APVM, GPM) extracted the following data about the characteristics and results of the included studies using an ad hoc data extraction form which had been piloted in advance: publication year, study design (e.g., randomized controlled trial), study location, setting, number of participants, aim of the study, type of breast cancer guideline (e.g., breast cancer screening), type of intervention (e.g., computerized decision support systems), and outcome(s) assessed (e.g., compliance rate). A second author (ENG, IRC LN, ZSP, EP, DC, APVM, GPM) cross-checked the extracted data for accuracy.

### Quality assessment

We used the following tools to determine the risk of bias of the included studies: the Cochrane Collaboration tool for assessing risk of bias in randomized trials (RoB I) [27], the ROBINS I tool for non-randomized controlled before-after studies [28], the Quality Assessment Tool for Before-After (Pre-Post) Studies With No Control Group [29], the Newcastle-Ottawa scale for cohort studies [30], the AXIS tool for cross-sectional studies [31], and the MMAT tool [32] for mixed methods studies. The specific criteria included by each of these tools are available in Additional file 3. One author determined the risk of bias of the included studies, and a second author cross-checked the results for accuracy. Disagreements were solved with support from a senior systematic reviewer.

### Data synthesis

We described the characteristics and the effects of the interventions narratively and as tabulated summaries. Findings are synthesized by type of intervention. We applied the Cochrane Effective Practice and Organization Care Review Group (EPOC) [33] taxonomy to classify our findings according to the types of interventions identified. Whereas for the characterization of the interventions we included all the publications identified meeting our eligibility criteria (irrespectively of their design); for the evaluation of the effectiveness of the interventions we focused only on those studies following a suitable design for such purpose [34]: randomized controlled trials (RCTs), controlled before-after studies, non-randomized controlled trials, and interrupted time series. Although we planned to conduct a meta-analysis on the impact of the interventions on compliance rates, this was finally not feasible due to the inconsistent and poor reporting. Instead, we provide a graphical quantitative description of the compliance rates before and after the implementation of the interventions.

### Certainty of the evidence

Following the GRADE approach [35], we rated the certainty of evidence as high, moderate, low or very low, taking into consideration risk of bias, imprecision, inconsistency, indirectness, and publication bias. This was done by one researcher. and cross-checked by a second reviewer.

## Results

### Search results

**Fig. 1:**
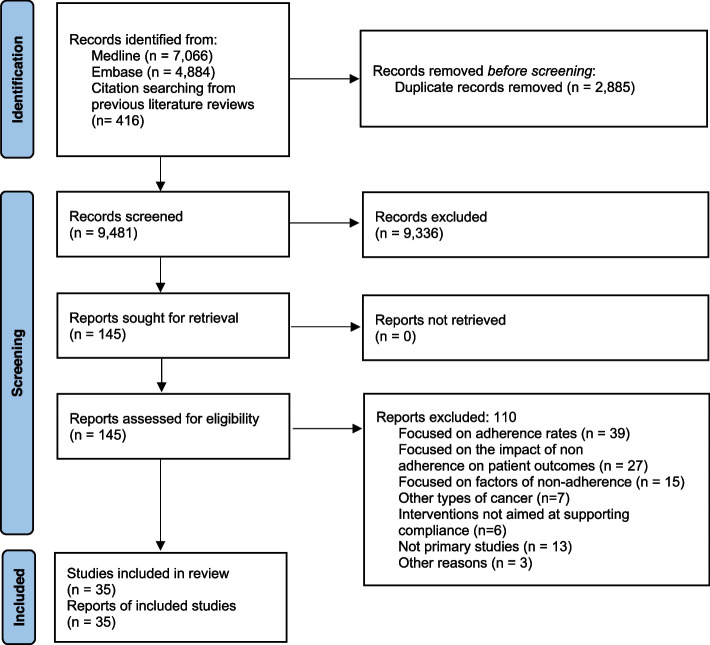
PRISMA flowchart

The eligibility process is summarized in a PRISMA flowchart (Fig. 1). We retrieved a total of 9065 unique citations from database searches, which were reviewed (through screening by title and abstract) along with 416 additional references identified from the thirteen systematic reviews also identified. We selected 145 references for full text revision, from which 35 primary studies (reporting on 24 different interventions) were finally included in our systematic review [36–70].

### Characteristics of the included studies

The characteristics of the included studies are summarized in Table 1 and described in detail in Additional file 4. Most (86%) were published from 2000 onwards. The studies were conducted in six countries: 15 (42%) were conducted in USA [37, 44–46, 49–51, 53–58, 60, 70], 12 (34%) in France [38–43, 63–68], 3 (9%) in the Netherlands [52, 62, 69], and 3 (9%) in Canada [36, 59, 61]. The remaining two studies were conducted in Australia [47], and Italy [48]. Eleven studies described interventions to support compliance with guidelines on diagnosis and treatment [41, 43, 52, 56, 64–70], 9 focused on treatment only [38–40, 42, 47–49, 62, 63], 5 on diagnosis only [45, 51, 58–60], and 7 on screening [36, 37, 46, 50, 54, 57, 61]. Six studies were randomized controlled trials [37, 45, 50, 51, 54, 60], four were non-randomized controlled trials [46, 57, 58, 63], eight non-controlled before-after studies [42, 49, 53, 55, 59, 62, 65, 69], one prospective cohort study , three cross-sectional studies [44, 47, 56], one mixed-methods [36] and twelve case studies [38–41, 43, 48, 52, 61, 64, 66–68].

**Table 1:** Characteristics of the included studies (*N*=35)

Study characteristics	*N* (%)	References
Country		
Australia	1 (3%)	[47]
Canada	3 (9%)	[36, 59, 61]
France	12 (34%)	[38–43, 63–68]
Italy	1 (3%)	[48]
Netherlands	3 (9%)	[52, 62, 69]
USA	15 (42%)	[37, 44–46, 49–51, 53–58, 60, 70]
Publication year		
2016–2021	6 (17%)	[36, 39, 44, 49, 53, 55]
2011–2015	11 (32%)	[38, 41, 43, 48, 56, 61, 66–70]
2006–2010	5 (14%)	[37, 50, 52, 59, 65]
2001–2005	8 (23%)	[40, 42, 46, 57, 60, 62–64]
< 2001	5 (14%)	[45, 47, 51, 54, 58]
Study design		
Randomized controlled trial	6 (17%)	[37, 45, 50, 51, 54, 60]
Controlled before after study	4 (11%)	[46, 57, 58, 63]
Non-controlled before-after study	8 (23%)	[42, 49, 53, 55, 59, 62, 65, 69]
Prospective cohort study	1 (3%)	[70]
Mixed-methods	1 (3%)	[36]
Cross-sectional	3 (9%)	[44, 47, 56]
Case study	12 (34%)	[38–41, 43, 48, 52, 61, 64, 66–68]
Type of guidelines‡		
Screening	7 (20%)	[36, 37, 46, 50, 54, 57, 61]
Diagnosis	5 (14%)	[45, 51, 58–60]
Treatment	9 (26%)	[38–40, 42, 47–49, 62, 63]
Diagnosis and treatment	11 (31%)	[41, 43, 52, 56, 64–70]
Surveillance/follow-up/rehabilitation	3 (9%)	[44, 53, 55]
Type of intervention		
Computerized decision support systems	12 (34%)	[38–43, 48, 64–68]
Educational interventions	7 (20%)	[44, 50, 55, 57–59, 63]
Multifaceted	9 (25%)	[36, 37, 46, 49, 51, 53, 54, 60, 62]
Audit and feedback	2 (6%)	[47, 69]
Clinical pathways	1 (3%)	[56]
Integrated knowledge translation	1 (3%)	[61]
Medical critiquing system	1 (3%)	[52]
Medical home program	1 (3%)	[70]
Provider reminders	1 (3%)	[45]
Outcomes assessed		
Intervention adoption/fidelity	2 (6%)	[36, 44]
Impact on providers attitudes/knowledge/self-efficacy	4 (12%)	[43, 46, 50, 57]
Impact on compliance with CPG recommendations	30 (85%)	[36–43, 45–51, 53–56, 58–60, 62, 63, 65–70]
Impact on patient health related outcomes	0 (0%)	–
Costs of implementing the interventions	1 (3%)	[44]
Risk of bias		
Low	5 (14%)	[45, 53, 54, 59, 70]
Moderate	10 (29%)	[36, 37, 42, 44, 47, 56–58, 62, 63]
High	5 (14%)	[46, 49, 50, 55, 65]
Unclear	4 (11%)	[51, 52, 60, 69]
Not assessed (case studies) ^a^	11 (31%)	[38–41, 43, 48, 61, 64, 66–68]

*N* number of studies. % percentage of studies

^a^Risk of bias not assessed in studies following a case study design

Thirty of the 35 studies (85%) evaluated the impact of the interventions on compliance rate [36–43, 45–51, 53–56, 58–60, 62, 63, 65–70]. Four studies [43, 46, 50, 57] evaluated the impact on determinants of behavior change related outcomes (providers’ knowledge, attitudes, and self-efficacy about the CPGs recommendations). Two studies evaluated intervention adoption and fidelity [36, 44]. No study evaluated the impact of the intervention on patient outcomes, and only one study [44] evaluated the costs of implementing the interventions.

### Characteristics of the interventions to support compliance with breast cancer clinical practice guidelines

Table 2 describes the characteristics of each type of intervention. Twelve studies described two different interventions consisting in the implementation of computerized decision support systems [38–43, 48, 64–68], 7 described 6 different educational interventions targeting health care professionals [44, 50, 55, 57–59, 63], 9 described 9 multifaceted interventions [36, 37, 46, 49, 51, 53, 54, 60, 62], and two studies described two audit and feedback interventions [47, 69]. The rest of the studies described interventions based on: implementation of clinical pathways [56], integrated knowledge translation systems [61], medical critiquing system [52], medical home program [70], and reminders to providers [45].

**Table 2:** Characteristics of the interventions identified to support healthcare providers´ compliance with guideline recommendations for breast cancer

Author(s)/publication year/reference	Brief name of the intervention	Where	Intervention goal Determinants/behaviors the intervention sought to change	Intervention description	Who provided	When and how much
A. *Computerized clinical decision support system*						
Seroussi, Bouaud et al. [38–43, 64–68]	OncoDoc/OncoDoc2	In hospitals in France	Intervention not targeting a specific behavior. Rather, to support state-of-the-art clinical decision making, by increasing physician awareness and knowledge of CPGs contents.	Guideline-based computer decision support system, providing patient-specific recommendations in the management of non-metastatic invasive breast cancer according to local guidelines (CancerEst). The system relies on a formalized knowledge base structured as a decision tree. Starting from the root of the decision tree, the physician user navigates through the knowledge base while answering questions and thus instantiating patient criteria. Guideline-based patient-centered therapeutic recommendations are provided when the navigation is completed, i.e., a leaf is reached	Computer system	Intervention available as part of routine clinical practice (healthcare professionals had unlimited access to it). The intervention was used during multidisciplinary staff meetings, to inform therapeutic decisions concerning cancer patients.
Eccher et al. 2014 [48]	OncoCure CDSS	Medical Oncology Unit, Hospital of Trento (Italy).	To support appropriate adjuvant medical treatment to BC patients during all the stages of therapy	Asbru-based decision support system implementing treatment protocols for breast cancer, which accesses data from an oncological electronic patient record.	Computer system	Two temporally distant groups of multidisciplinary panel discussions: 36 cases were discussed in meetings held in the last third of 2009, 25 cases were discussed in meetings held in the first third of 2012
*B. Provider educational intervention*						
Gorin et al. 2006 [50]	Not reported	Primary care centers in underserved community in New York (USA)	To increase mammography referrals in community-based urban physicians	Physician-directed education, academic detailing, using the American Cancer Society guidelines for the early detection of BC. Self-learning packets (i.e., professionally designed print materials, scientific articles, and a targeted verbal script)	Two Master’s level health educators	4 academic detailing visits over a 2-year period of time. Academic detailing contacts were brief (average, 9.25 min). If the physician consented, the office-based breast cancer prevention materials were shared with the other staff as well. Visits were supplemented by 6 dinner seminars.
Lane et al. 1991 [58]	Not reported	Primary care centers in New York (USA). Although the target were primary care centers, most of the intervention was delivered at the local community hospital.	To increase mammography referrals and physical breast examination for women 50 years of age and older.	Multimethod approach to physician education including conferences, physician newsletters, skills training, BC monograph, “question of the month” among hospital staff meetings, primary care office visits and patient education materials.	Research team	Intervention delivered over 2 years
Ray-Coquard et al. 2002 [63]	Not reported	Hospitals in France (ONCORA cancer network)	Intervention not targeting a specific behavior, but rather to increase overall compliance by increasing physicians’ knowledge about the guidelines in place.	Monthly meetings where the relevant sections of the CPGs were presented. The information was then discussed, modified and/or validated by all the participating physicians from the hospitals to obtain a regional consensus. Two weeks after the meeting, the validated CPGs were sent to all the participating physicians who were expected to use them in their practice. No specific penalty or reward system was included in this implementation strategy.	Local opinion leaders (from the cancer center), who were both knowledgeable and credible for the specific cancer site.	Monthly meetings, taking place in 1995.
Lane et al. 2001 [57]	Not reported	Primary care centres in NewYork (USA).	To increase mammography referrals	1–2 h in-office training program and/or self-study workbook according to physician CME need. The CME intervention was offered as a comprehensive, multifaceted package.	Master’s level nurse educator and standardized patient (trained to provide feedback to the physicians to enhance their clinical breast examination and communication skills).	1–2 h training session (tailored according to each physician's pre-intervention level of adherence to breast cancer screening guidelines and/or CME need)
Kreizenbeck et al. 2020 [55]	Not reported	Regional community oncology clinics in Washington (USA)	Improve prescription in relation to the use of primary prophylactic colony stimulating factors for chemotherapy regimens with < 20% risk of febrile neutropenia	Academic detailing for oncologists at a regular meeting	Not reported	1 session
McWhirter et al. 2007 [59]	Not reported	Cancer center in Toronto (Canada)	To reduce the number of unnecessary staging investigations performed	Multidisciplinary educational rounds, highlighting the Cancer Care Ontario Practice Guidelines, and reporting results of the audit of staging investigations. Attendance at the rounds included staff and trainees in medical and radiation oncology, surgical oncology and pathology. The guidelines were widely distributed to the medical, surgical and radiation oncologists, in a hard copy format.	Not reported	January–March 2003
Calo et al. 2020 [44]	Strength after Breast Cancer (SABC)	Outpatient rehabilitation clinics in the USA	To improve knowledge about how to deliver evidence-based rehabilitative exercise interventions for breast cancer survivors	The online course was provided through a popular online platform for physical therapy continuing education (Klose Training and Consulting website; http://klosetraining.com/course/online/strength-abc). The covered all aspects of setting up and running the SABC program including how to obtain referrals from oncology clinicians, screen potential patients, coordinate with a certified lymphatic therapist, educate patients about lymphedema, teach the 4-session exercise program, instruct patients on how to log their progress, motivate patients to perform exercises, handle logistical considerations, and manage discharge and wrap-up. The course also provided all the materials needed to set up the program in clinics.	Online course prepared by the researchers	The online course was provided in 2015. The course was 4-h long.
*C. Audit and feedback interventions*						
Veerbeek et al. 2011 [69]	Not reported	Hospitals in the Midwestern part of the Netherlands	To improve the diagnostic process and surgical treatment for women with breast cancer	Written report with regional benchmark information on nine performance indicators measuring quality of care based on BC National Guidelines. The intervention was based on the Plan-Do-Study-Act cycle for continuous quality improvement. Each year from 2002 until 2006, hospitals received a written report with regional benchmark information on each indicator. Furthermore, in 2004, 2005, and 2006, the care professionals attended training sessions twice a year. During these training sessions, an anonymous benchmark was presented in which the indicator scores for each hospital were compared with the regional mean score and the norm score. The care professionals generally discussed those indicator scores that clearly deviated from the regional mean score or the norm score with experts in the field. In 2006, 2007, and 2008, a member of the multidisciplinary team presented the benchmark information to the Oncology Committee within each hospital. This presentation for direct colleagues stimulated the care professionals to discuss the results more freely and to initiate improvement initiatives.	Research team	Intervention delivered from 2002 to 2006 (two sessions per year)
Craft et al. 2000 [47]	Not reported	Breast cancer treatment facilities and medical practices in Australia	Increase awareness and knowledge about guideline treatment recommendations for breast cancer	Audit of healthcare provided to patients (based on medical records) according to four indicators. Data was fed back to each participating clinician, providing comparisons across the group and against the agreed criteria. Aggregated data across the whole clinician group were presented at regular meetings of the treatment group	Research team	May 1997 to July 1998
*D. Multifaceted interventions*						
Aspy et al. 2008 [37]	Not reported	Primary care practices in Oklahoma (USA)	To increase mammography referrals in women over 40	Multicomponent intervention, which included audit and feedback; academic detailing of exemplar principles and information from the medical literature; services of a practice facilitator; and information technology support. Practices were free to choose (or not) from the identified exemplar strategies or to modify them as necessary to fit the practice constraints of their individual settings.	Researchers developed the materials. A trained practice facilitator spent at least 2 days per month at each practice and helped the practitioners design their interventions and facilitate the “Plan, Do, Study, Act” process.	Intervention delivered during 9 months
Michielutte et al. 2005 [60]	Not reported	Primary care practices in North Carolina (USA)	To increase mammography referrals among women over 65	The intervention program was based on two theoretical models: Health Belief Model, and the Transtheoretical or Stages of Change model. The intervention consisted on: provider education (information on issues in mammography for older women); written educational materials on BC and screening mailed to women; and a brief telephone counseling session for the women.	Researchers	The intervention lasted approximately 9 months. The intervention design was sequential, with progressively more intensive interventions introduced at each stage.
Hillman et al. 1998 [54]	Not reported	Primary care practices in Philadelphia (USA)	To increase screening mammography among women over 50	Semi-annual feedback to primary care providers regarding compliance with cancer screening guidelines and financial bonuses for "good" performers. • Feedback reports documented a site’s scores on each screening measure and a total score across all measures, as well as planwide scores for comparison. Bonuses ranged from $570 to $1260 per site, with an average of $775 per audit. Seventeen (of 26) sites received at least 1 bonus throughout the course of the study	Researchers	Intervention took place from 1993 to 1995. Chart audits were performed at baseline and every 6 months for 1.5 years.
Grady et al. 1997 [51]	Not reported	Small, primary care practices in Massachusetts (USA)	Increasing mammography referrals by primary care physicians	• Physician education: included discussion of charts illustrating historical breast cancer incidence the rising number of older women in the population, the strong association of breast cancer and age, and the relationship between physician encouragement and mammography use. • Cue enhancement: two kinds of cues supplemented the educational material. General cues were posters provided for waiting or treatment rooms, chosen to emphasize breast cancer risk among older women and the efficacy of mammography. Specific cues were chart stickers in a schematic breast shape with spaces for recording three mammography referrals and completions. • Feedback rewards: peer-comparison feedback about mammography use and token monetary rewards. Individualized feedback was provided in two charts that were mailed to each physician in the practices.	Researchers	1 year intervention (unclear dates). Education: one session Feedback: four feedback reports sent quarterly. Financial incentive: check based on the percentage referred during each audit period (i.e., $50 for a 50% referral rate).
Gilbo et al. 2018 [49]	Not reported	Hospitals in USA	Support the proper use selection of hypofractionation of breast irradiation (which was underutilized)	Five consensus-driven and evidence-based clinical directives to guide treatment decisions were implemented. Prospective contouring rounds were instituted, wherein the treating physicians presented their directive selection and patient contours for peer-review and consensus opinion.	Working committee that consisted of physicians, dosimetrists, nurses, and physics and therapy staff	Directives became available for its use as part of routine clinical practice in 2010
Hill et al. 2018 [53]	Not reported	Health facilities part of the Gundersen Medical Foundation, including 30 regional clinics and 5 rural hospitals (USA)	To decrease laboratory testing for early breast cancer patients	• Provider education: PowerPoint presentations were delivered at 2-month intervals, where changes in breast cancer guidelines for testing were cited. • Audit and feedback: peer performance comparisons (benchmarking) with full transparency to providers and tumor board attendees by disclosing individual ordering provider performance compared to others. • Certification: specific questions that would qualify for continuing medical education credits. • Patient education: Information fact sheets containing information on the guidelines were created for patient education at their initial appointment. In these, patients would be encouraged to discuss lab testing and imaging with their provider • Financial incentives: To reward and incentivize providers, a plan was discussed to reward them for high guideline compliance with gift certificates to local restaurants. • Health information technology: Implementation of alerts in the electronic medical records.	• Provider education: delivered by surgical resident-in-training, a medical student, and the principal investigator. • Audit and feedback: by academic researcher	• Provider education: delivered at 2-month intervals beginning June 2016 • Audit and feedback: delivered in October 2016 and January 2017
Ottevanger et al. 2004 [62]	Not reported	Hospitals part of the Comprehensive Cancer Centre (Netherlands)	• To support the provision of treatment according to a guideline for premenopausal node-positive breast cancer patients	• Audit and feedback: repeated feedback on the performance of the chemotherapy administration, timing and dosing was delivered through of oral presentations, during three breast cancer group meetings. • Educational activities: Important literature that became available in that period on the dose intensity of chemotherapy, sequencing of radiotherapy and the importance of adequate axillary lymph node clearance were discussed	• Researchers	• Audit and feedback: Between 1993 and 1996. • Educational activities: four consecutive meetings
Coleman et al. 2003 [46]	Not reported	Primary care clinics in Arkansas (USAa)	To increase breast cancer screening among low-income, African American, and older women (increasing healthcare professionals’ knowledge to improve encouragement of screening)	• Use of standardized patients to observe and record providers’ performance followed by direct feedback: standardized patient was a lay woman trained in a particular clinical scenario to score and teach CBE using herself as a model. • Newsletters to inform providers about screening methods: four newsletters in a format that was easy to read and clinically relevant for busy healthcare professionals in primary care. They provided the latest information about breast cancer screening, diagnosis, treatment, and rehabilitation. • Posters and cards presenting key points about CBE and the importance of screening mammograms: Two sets of posters presenting key points about CBE and the importance of routine screening mammograms, along with laminated pocket-size cards with the same information, were provided to the clinics in the intervention group to display. The posters were designed to prompt women to discuss screening issues with providers • Patient education materials about breast cancer screening available from the National Cancer Institute and American Cancer Society were mailed to clinics	Research team	May 1996–June 1998
Armson et al. 2018 [36]	Not reported	Primary care practices in Canada	Increasing mammography referrals by primary care physicians	Set of iTools for patients and clinicians: • Screening Recommendations for breast cancer with mammography (printed educational material targeted to clinicians) • Screening recommendations for clinical breast exams and breast self-exams (printed educational material targeted to clinicians) • Breast cancer online video: targeted to clinicians, exploring strategies for patient discussion around breast cancer screening issues • Patient handout: “*breast cancer screening—what is the right choice for me?”* patient decision aid • Patient handout: printed educational material with algorithm guides individuals re mammography • Patient handout: Patient handout describing benefits and the risks of breast cancer for women between 40 and 49, 50–69, and 70–74 years of age. It includes a pictorial representation of outcome of screening in each age group including false positives, biopsy, and mastectomy • Patient handout: printed educational material highlighting that CTFPHC recommends that women aged 50–74 schedule a mammogram every 2–3 years	Researchers/ Canadian Task Force on Preventive Health	September 2013 to august 2013
*E. Other types of interventions*						
Chambers et al. 1989 [45]	Not reported	Primary care practices in the USA	Increasing mammography referrals by primary care physicians	***Provider reminders*** Microcomputer tickler system for ordering of mammograms. The date of the last mammogram ordered and entered into the database was displayed in the comments section of the encounter form for each visit. This information was printed as “last mammogram: date”, or, if no mammogram was on record in the encounter form database, the notation was listed as “last mammogram: ?” Entering a physician-ordered mammogram into the database automatically updated the reminder in the comments section for subsequent visits.	Computer system	November 1986 (system available during 6 months)
Wheeler et al. 2013 [70]	Community Care of North Carolina (CCNC) Program	Primary care practices in the USA	To support the provision of guideline-concordant follow-up care among breast cancer survivors.	***Medical home program*** Innovative medical home program to enhance primary care case management in vulnerable populations insured by Medicaid. Medicaid patients whose providers are members of one of the CCNC networks throughout the state are enrolled into a CCNC medical home, and their providers and the network receive per member per month payments for care coordination.	Health system	This medical home program became available in 1990 as part of routine care organization for eligible centres.
Kubal et al. 2015 [56]	Not reported	Moffitt Cancer Center (USA)	To overcome the challenges of adherence to clinical pathways	***Computerized vignettes*** Computerized vignettes that simulate patient scenarios and ask clinicians to make decisions. The vignettes consisted of 12 different cases that required the provider to evaluate a simulated female patient for breast cancer or suspected breast cancer.	Researchers	March 2013 onwards. Providers completed 2 vignettes every 4 months for 6 rounds over a period of 20 months. Each vignette takes approximately 25 min to complete
Munce et al. 2013 [61]	Not reported	Not implemented	Facilitate the uptake of the breast cancer screening guidelines	***Integrated knowledge translation intervention*** Integrated knowledge translation strategy based on the Knowledge to Action framework: seven action cycle phases to guide the development of a strategy to implement this knowledge (guidelines) in healthcare settings. The suggested strategy resulting from the use of the framework targeted providers and patients, and included: • Continuous medical education event targeted to physicians managers • Point-of-care tools such as CDS tools, computer order-entry systems • (reflecting new guidelines) • Decision aids targeted to patients (through internet, patient decision groups, magazines)	Intervention developed by an interprofessional group of students as well as two faculty members met six times over three days at the KT Canada Summer Institute in 2011	Not applicable (intervention designed but not implemented)
Groot et al. 2009 [52]	Not reported	Dutch Comprehensive Cancer Centre (Netherlands)	To improve overall compliance with breast cancer clinical guidelines	***Medical critiquing system*** Automated system to compare clinical actions performed by a physician with a predefined set of actions. The results were fed back to clinicians.	Computer system	January 2003—June 2004

*CDSS* Computerized decision support systems, *CME* Continuing medical education, *CPG* Clinical practice guideline

#### Computerized decision support systems

The use of computerized decision support systems to promote compliance with breast cancer CPGs was described in 12 studies [38–43, 48, 64–68]. Eleven of them reported the same intervention, which consisted of a system developed in France called OncoDoc [38, 40–43, 64–68). OncoDoc is a computerized clinical decision support system that provides patient-specific recommendations for breast cancer patients according to CancerEst (local) CPGs [71]. A study conducted in Italy reported on the development of a similar system, the OncoCure CDSS [48].

#### Educational interventions

Seven studies described educational interventions targeting healthcare providers to promote compliance with breast cancer CPGs [44, 50, 55, 57–59, 63]. One intervention consisted in the provision of academic detailing on breast cancer screening (based on the American Cancer Society guidelines for the early detection of BC) among primary care physicians in an underserved community in the USA [50]. An intervention in seven hospitals in France consisted in monthly meetings where local opinion leaders presented the relevant sections of the CPGs, which were subsequently sent to all the participating physicians who were expected to use them in their practice [63]. Another intervention consisted in a comprehensive continuing medical education package to address pre-identified barriers to guideline adherence. The intervention followed a multimethod approach to physician education including CME conferences, physician newsletters, CBE skills training, BC CME monograph, “question of the month” among hospital staff meetings, primary care office visits, and patient education materials [57, 58]. An educational intervention to improve compliance with radiological staging CPGs in early breast cancer patients [59] consisted of multidisciplinary educational rounds, presenting the Cancer Care Ontario Practice Guidelines [72]. Another intervention, aimed to support compliance with recommendation against serum tumor marker tests and advanced imaging for BC survivors who are asymptomatic for recurrence, consisted in academic detailing for oncologists at regular meetings [55]. Another intervention [44] consisted in an online course to learn to implement and deliver the Strength after Breast Cancer (SABC) guidelines (with recommendations about rehabilitative exercise for breast cancer survivors).

#### Audit and feedback interventions

We identified two audit and feedback interventions [47, 69]. One consisted in sending hospitals a written report with regional benchmark information on nine performance indicators measuring the quality of care based on breast cancer national guidelines [69]. Healthcare professionals attended sessions twice a year, where an anonymous benchmark was presented for each hospital score compared with the regional mean and the norm scores. Another intervention [47] audited patients’ medical records according to four agreed indicators. Information from the audit forms was entered into a database, which allowed individualized reports for each participating clinician, providing detailed feedback about their practice, with comparisons across the group and against the agreed criteria.

#### Other types of single component interventions

Five studies described other strategies to promote compliance with breast cancer CPGs [45, 49, 52, 56, 61, 70]. One intervention consisted on a microcomputer tickler system on the ordering of mammograms [45]. The system displayed the date of the last mammogram ordered in the “comments” section of the encounter form for each visit. An intervention to support compliance with CPGs follow up recommendations in low-income breast cancer survivors [70] consisted in the implementation of a medical home program to support primary care case management. Providers and networks participating in this program received a payment per eligible patient per month for care coordination. Another intervention consisted in the implementation of new clinical pathways supplemented by clinical vignettes [56]. Another intervention consisted in an integrated knowledge translation strategy to be used by guideline developers to improve the uptake of their new CPGs on breast cancer screening [61]. This integrated knowledge translation strategy was based on the Knowledge to Action Framework [73], and involved the identification of barriers to knowledge use. An intervention to support compliance with the Dutch breast cancer guideline [52] consisted of a medical critiquing system (computational method for critiquing clinical actions performed by physicians). The system aimed at providing useful feedback by finding differences between the actual actions and a set of ‘ideal’ actions as described by a CPG.

#### Multifaceted interventions

We identified nine multifaceted interventions [36, 37, 46, 49, 51, 53, 54, 60, 62]. One intervention to increase compliance with mammography screening [37] consisted of (i) audit results and a comparison with the network benchmark; (ii) academic detailing of exemplar principles and information from the medical literature; (iii) services of a practice facilitator for 9 months who helped the practitioners design their interventions and facilitate “Plan, Do, Study, Act” processes; and iv) information technology support. In another intervention [60] to increase screening mammography, primary care providers received (i) a fact sheet providing current information on screening mammography for older women; (ii) telephone follow-up of any questions, and; (iii) copies of a simply written pamphlet on mammography that they could distribute to patients. Another intervention [54] consisted of biannual feedback to primary care providers regarding compliance with cancer screening CPGs and financial bonuses for “good” performers. Feedback reports documented a site’s scores on each screening measure and a total score across all measures, as well as plan-wide scores for comparison. Another intervention [51] consisted of an educational intervention accompanied by cue enhancement using mammography chart stickers, and by feedback and token rewards. Another intervention [46] included (i) use of standardized patients to observe and record healthcare professionals’ performance followed by direct feedback; (ii) newsletters to inform healthcare providers about screening methods; (iii) posters and cards presenting key points about CBE and the importance of routine screening mammograms, and; (iv) patient education materials. An intervention to improve compliance with new CPGs by the American Society for Radiation Oncology (ASTRO) on the proper use of hypofractionation [49] consisted in implementing five consensus-driven and evidence-based clinical directives to guide adjuvant radiation therapy for breast cancer. Prospective contouring rounds were instituted, wherein the treating physicians presented their directive selection and patient contours for peer-review and consensus opinion. Another intervention combined audit and feedback and education to providers to increase compliance with breast cancer treatment guidelines [62]. Repeated feedback on the performance of the chemotherapy administration, timing and dosing were given to the participants. The feedback consisted of a demonstration of variation in performance between the different hospitals and the region as a whole. The educational component consisted in four consecutive sessions of discussion about relevant literature that became available in that period regarding chemotherapy dose intensity, sequencing of radiotherapy and the importance of adequate axillary lymph node clearance.

An intervention to promote compliance with new National Comprehensive Cancer Network guidelines from routine testing to omission of ordering complete blood cell count and liver function tests in patients with early breast cancer [53] involved (i) provision of educational materials; (ii) audit and feedback; (iii) certification; (iv) patient education; (v) financial incentives and (vi) implementation of alerts in the electronic medical records. Another intervention to promote breast cancer screening CPGs [36] included (i) printed educational materials with the recommendations for breast cancer mammography, (ii) printed educational materials with CPGs recommendations for clinical breast exams and breast self-exams, and (iii) video (12 min) directed at clinicians, exploring strategies for patient discussion around breast cancer screening issues.

### Risk of bias

The risk of bias was judged as low in five studies [45, 53, 54, 59, 70], moderate in ten [36, 37, 42, 44, 47, 56–58, 62, 63], and high in five [46, 49, 50, 55, 65]. In four studies [51, 52, 60, 69] the risk of bias was unclear since there was not enough information available to determine potential biases. We did not assess risk of bias for case studies, due to the lack of appropriate tools available. A detailed description of the risk of bias of the included studies, excluding case studies, is available in Additional file 3.

### Impact of the interventions

**Fig. 2:**
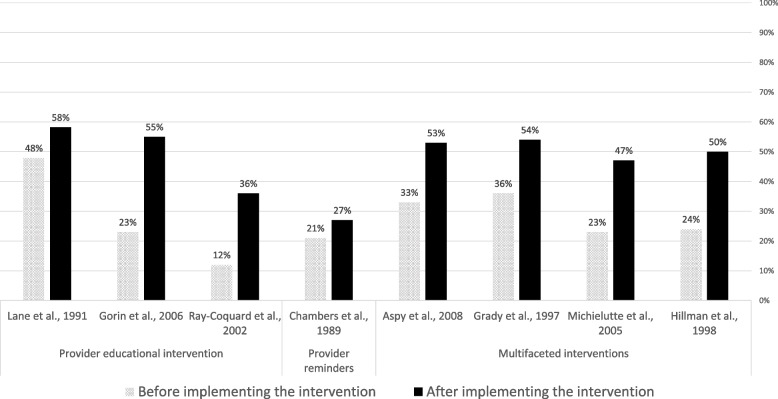
Compliance rate with guideline recommendations before and after the implementation of the identified interventions

Six RCTs [37, 45, 50, 51, 54, 60] and four controlled before-after studies [50, 57, 58, 63] examined the effectiveness of four provider educational interventions, one intervention based on the use of provider reminders, and five multifaceted interventions. In nine of these interventions (90%), the ultimate goal was to improve compliance with breast cancer screening guidelines. Compliance was uniformly measured in terms of mammography rates (e.g., proportion of eligible women undergoing a mammography screening for breast cancer). Except one multifaceted intervention [54], the interventions consistently showed relevant beneficial effects (Fig. 2).

#### Impact of educational interventions

Four studies evaluated the effectiveness of educational interventions targeted to healthcare providers [50, 57, 58, 63]. A randomized controlled trial showed that the intervention improved recommendation of mammography (odds ratio (OR) 1.85, 95% CI 1.25–2.74) and clinical breast examination (OR 2.13, 95% CI 1.31–3.46) in female patients aged 40 and over [50]. One controlled before-after study showed significant (*p* < 0.05) improvements in providers’ knowledge, attitudes and self-efficacy towards the new CPG screening recommendations [57], whereas another controlled before-after study reported a significant improvement in the number of reported mammography referrals of asymptomatic women aged 50 to 75 years in the intervention group but not in the control group [58]. A controlled before-after study observed an improved compliance to diagnostic and treatment CPG recommendations in the intervention group (from 12% before the intervention to 36% post-intervention; *P* < 0.001), whereas no significant improvements were observed in the control group [63].

#### Impact of provider reminders

A randomized controlled trial [45] showed that a microcomputer-generated reminder system for ordering mammograms improved compliance with mammography guidelines: 27% (170/639) in the intervention vs 21% (128/623) in the control group (OR = 1.40 (95%CI = 1.01 to 1.82); *p* = 0.011) after 6 months follow-up.

#### Impact of multifaceted interventions

Five studies examined the impact of multifaceted interventions. A randomized controlled trial observed that, in comparison with usual care, a multifaceted intervention (including audit and feedback; provider education; information technology support) increased the proportion of women offered a mammogram (38% vs 53%), and the proportion of women with a recorded mammogram (35% vs 52%) [37]. Another trial observed that a multifaceted intervention (comprising provider education and patient education through pamphlets), did not improve compliance with screening mammography guidelines in the overall sample, but produced significant improvements in specific vulnerable subgroups (elderly, lower educational attainment, black ethnicity and with no private insurance) [60]. A randomized controlled trial observed that a multifaceted intervention (audit and feedback plus financial incentives) doubled screening rates both in the intervention and control groups, with no statistically significant differences observed between groups [54]. A trial examining a multifaceted intervention (provider education, cue enhancement plus feedback, and token rewards) observed that mammography compliance rates significantly improved (*p* < 0.05) in the intervention (62.8%) in comparison with the control (49.0%) group [51]. A controlled before-after study observed that a multifaceted intervention (including audit and feedback, patient and professional education) improved the demonstration of breast cancer screening, with significantly more women older than 50 receiving mammograms in the intervention than in the comparison group [46].

#### Certainty of evidence

The results from the assessment of the certainty of evidence concerning the impact of the interventions on compliance with breast cancer CPGs is available in Additional file 5. Based on GRADE criteria, we rated the certainty of evidence as “low” for the four educational interventions targeting healthcare providers. This was due to very serious risk of bias, for which we downgraded the level of evidence two levels. For the only intervention identified consisting in a reminder system for healthcare providers, we rated the certainty of evidence as “moderate” (downgrading one level due to serious indirectness). For the five multifaceted interventions, we rated the evidence as “low”, due to serious risk of bias, and serious inconsistency.

## Discussion

### Main findings

In this systematic review, we identified 35 studies describing and evaluating the impact of interventions to support clinician compliance with breast cancer CPGs. We described a range of different types of interventions to support adherence of healthcare professionals to breast cancer CPGs. We observed that there is low quality evidence that educational interventions targeted at healthcare professionals may improve compliance with recommendations concerning breast cancer screening, diagnosis and treatment. There is moderate quality of evidence that reminder systems for healthcare professionals improve compliance with recommendations concerning breast cancer screening. There is low quality of evidence that multifaceted interventions may improve compliance with recommendations concerning breast cancer screening. The effectiveness of the remaining types of interventions identified is uncertain, given the study designs available (e.g., cross-sectional, uncontrolled before-after or case studies). There is very limited data on the costs of implementing these interventions.

### Strengths and limitations

The main strength of this systematic review is that it addressed a highly relevant question, and provided much needed evidence to help improve providers’ compliance with breast cancer guidelines globally. An additional strength is that, contrary to previous systematic reviews, ours was not limited to experimental studies. By including observational, and qualitative and mixed-methods studies, we were able to provide a richer characterization of the available interventions.

This review has several limitations. First, we restricted the bibliographic searches to peer-reviewed publications in English language only. This may have resulted in failing to identify additional relevant data that could have further informed our assessments of the available evidence. However, we think that the impact of this limitation is likely to be small, as suggested by a recent meta-epidemiologic study [74]. Second, the heterogeneity of the reporting of outcome data made meta-analysis not feasible. Third, the heterogeneity in outcomes and the large number of strategies used across studies precluded us to determine the unique influence of each strategy on a given outcome.

### Our results in the context of previous research

An important finding of our review is that most of the included studies showed that the interventions were effective in improving compliance to CPGs. This is in line with findings from previous, non-condition-specific reviews, which concluded that guideline dissemination and implementation strategies are likely to be efficient [75, 76].

A large proportion of the studies included in our review examined the impact of Computerized Decision Support Systems (CDSS). Previous systematic reviews observed that CDSS significantly improve clinical practice [77, 78]. In our review, the evidence about CDSS was only available from observational, uncontrolled studies, and was restricted to two tools in France and Italy in the hospital setting. New studies evaluating other CDSS, and in other settings and countries, are therefore needed.

There is substantial evidence from non-condition specific research that audit and feedback interventions can effectively improve quality of care [79]. A recent systematic review [80] examining the effectiveness of cancer (all types) guideline implementation strategies showed that providing feedback on CPG compliance was associated with positive significant changes in patient outcomes. More research is needed about the impact of audit and feedback interventions on the compliance with breast cancer CPGs.

Educational interventions targeted to providers (both in isolation and in combination with other interventions) have shown to improve outcomes in patients with cancer [80]. Despite the low certainty obtained, the studies in our review consistently showed that educational and multifaceted interventions improve compliance with breast cancer CPGs, supporting also results from previous non-condition specific reviews [16, 81], as well as current recommendations from the Institute of Medicine [82].

In line with our finding concerning electronic reminder interventions, a Cochrane systematic review concluded that computer‐generated reminders to healthcare professionals probably improves compliance with preventive guidelines [83].

### Implications for practice and research

In terms of implications for practice, given that compliance with breast cancer guidelines is associated with better survival outcomes [25], and that there are still a substantial proportion of breast cancer patients not receiving clinical guidelines recommended care [21], it is important that the most effective interventions available are implemented to improve breast cancer guideline uptake by healthcare providers.

In terms of implications for research, as in a previous non-condition-specific review [76], we observed that there is very limited data on the costs of implementing the interventions to support compliance with breast cancer CPGs, as well as a scarcity of studies evaluating the effectiveness of interventions targeting the organization of care (e.g., benchmarking tools). Research in these two areas is urgently needed to allow evidence-based decisions concerning which interventions should be rolled out and implemented widely as part of existing quality improvement programs. Also worth noting is that, up to now, the great majority of the research on this (breast cancer) area has focused on measuring the impact of the interventions on process measures (mostly compliance rates). No study measured the impact on patient outcomes, and only a small minority examined the impact on determinants of compliance behavior (e.g., providers’ knowledge, attitudes, or self-efficacy). Future research would benefit from including a broader range of outcomes (including proximal and distal), as this would help to better measure and understand the extent to which the interventions produce the intended benefits.

Future research is also needed to identify the most effective types of interventions in improving CPGs uptake, as well as the “active ingredients” of multifaceted interventions [84]. The characteristics of the CPGs intended users, and the context in which the clinical practice occurs are likely to be as important as guideline attributes for promoting adoption of CPG recommendations. Therefore, future research should focus on gaining a deeper understanding about how, when, for whom, and under which circumstances the interventions identified can effectively support guideline adherence. Using a realist evaluation methodology [85] may prove a valuable strategy in this endeavor. However, as observed in our review, the detailed characteristics of the interventions are very frequently scarcely reported. To allow progress in this area, it is of utmost importance that intervention developers and researchers offer in their published reports a comprehensive characterization of their interventions. The Template for Intervention Description and Replication (TIDieR) guidelines [86] were specifically designed for this purpose.

## Conclusion

Promoting the uptake and use of CPGs at the point of care, represents a final translation step, from scientific findings into practice. In this review we identified a wide range of interventions to support adherence of healthcare professionals to breast cancer CPGs. Most of them are based on computerized decision support systems, provision of education, and audit and feedback, which are delivered either in isolation or in combination with other co-interventions. The certainty of evidence is low for educational interventions. The evidence is moderate for automatic reminder systems, and low for multifaceted interventions. For the rest of the interventions identified, the evidence is uncertain. Future research is very much needed to strengthen the available evidence base, concerning not only their impact on compliance, but also on patient important outcomes, and on their cost-effectiveness.

## Supplementary Information

**Additional file 1.**  PRISMA 2020 Checklist.

**Additional file 2.**  Search strategy.

**Additional file 3.**  Summary of Risk of Bias Assessment.

**Additional file 4.**  Characteristics and results of the 35 studies included in the review.

**Additional file 5.**  Evidence Profiles.

## Data Availability

All data generated or analyzed during this study are included in this published article.

## Abbreviations

ASTRO: American Society for Radiation Oncology
CDSS: Computerized Decision Support Systems
CPG: Clinical Practice Guidelines
EPOC: Effective Practice and Organization Care
HR: Hazard ratio
OR: Odds ratio
PRISMA: Preferred Reporting Items for Systematic Reviews and Meta-Analyses
RCT: Randomized controlled trial
RoB: Risk of bias
SABC: Strength after Breast Cancer
TIDieR: Template for Intervention Description and Replication
