# Supplementary material for: Characteristics and impact of interventions to support healthcare providers’ compliance with guideline recommendations for breast cancer: a systematic literature review
Source: Implement Sci. 2023 May 22;18:17. doi: 10.1186/s13012-023-01267-2 (PMC10201699; doi:10.1186/s13012-023-01267-2)
Supplement: Supplementary file 2 — Additional file 2. Search strategy. [file 13012_2023_1267_MOESM2_ESM.docx]

**Additional file 2. Search strategy**

| ***Systematic reviews*** | |
| --- | --- |
| **MEDLINE**  PubMed  May 2019 | #1   “Guideline Adherence”[Mesh]  #2     adherence[ti]  #3     implement*[ti]  #4     uptake[ti]  #5     complian*[ti]  #6     #1 OR #2 OR #3 OR #4 OR #5  #7     guideline*[tiab]  #8     recommendation*[ti]  #9      #7 OR #8  #10    #6 AND #9  #11   systematic[sb]  #12   framework*[tiab]  #13   #11 OR #12  #14    #10 AND #13 |
| **EMBASE**  Ovid Embase  May 2019 | #1     adherence.ti.  #2     implement*.ti.  #3     uptake.ti.  #4     complian*.ti.  #5     #1 or #2 or #3 or #4  #6     guideline*.ti,ab.  #7     recommendation*.ti.  #8     #6 or# 7  #9     #5 and #8  #10     limit #9 to "systematic review"  #11     framework*.ti,ab.  #12     #9 and #11  #13     #10 or #12 |
| **Primary studies** | |
| **MEDLINE**  PubMed | #1  “Guideline Adherence”[Mesh]  #2  adherence[ti]  #3  implement*[ti]  #4  uptake[ti]  #5   complian*[ti]  #6    #1 OR #2 OR #3 OR #4 OR #5  #7  guideline*[tiab]  #8   recommendation*[ti]  #9    #7 OR #8  #10    #6 AND #9  # 11 “Neoplasms”[Majr]  #12  cancer[tiab]  #13  oncolog*[tiab]  #14  mammogram*[tiab]  #15  breast[ti] AND screen*[ti]  #16   #11 OR #12 OR #13 OR #14 OR #15  #17   #10 AND #16 |
| **EMBASE**  Ovid Embase  May 2019 | #1     adherence.ti.  #2     implement*.ti.  #3     uptake.ti.  #4     complian*.ti.  #5     #1 or# 2 or #3 or #4  #6     guideline*.ti,ab.  #7     recommendation*.ti.  #8     #6 or #7  #9     #5 and 8  #10     *neoplasm/  #11     cancer.ti,ab.  #12     oncolog*.ti,ab.  #13     mammogram*.ti,ab.  #14     (breast adj4 screen*).ti  #15     #10 or #11 or #12 or #13 or #14  #16     #9 and #15 |
