# Supplementary material for: Characteristics and impact of interventions to support healthcare providers’ compliance with guideline recommendations for breast cancer: a systematic literature review
Source: Implement Sci. 2023 May 22;18:17. doi: 10.1186/s13012-023-01267-2 (PMC10201699; doi:10.1186/s13012-023-01267-2)
Supplement: Supplementary file 3 — Additional file 3. Summary of Risk of Bias Assessment. [file 13012_2023_1267_MOESM3_ESM.docx]

**Additional file 3. Summary of Risk of Bias Assessment**

Randomized clinical trials

| **Cochrane RoB Tool** | | **Aspy 2008** | **Chambers 1989** | **Gorin 2006** | **Grady 1997** | **Hillman 1998** | **Michielutte 2005** |
| --- | --- | --- | --- | --- | --- | --- | --- |
| 1 | Random sequence generation | Low | Low | High | Low | Low | ? |
| 2 | Allocation concealment | ? | ? | High | Low | ? | ? |
| 3 | Blinding of participants and personnel | High | Low | High | ? | Low | Low |
| 4 | Blinding of outcome assessment | ? | Low | Low | ? | Low | ? |
| OVERALL RATING | | MODERATE | LOW | SERIOUS | UNCLEAR | LOW | UNCLEAR |

Controlled before-after studies

| **ROBINS I Tool** | | **Coleman 2003** | **Lane 1991** | **Lane 2001** | **Ray-Coquard 2002** |
| --- | --- | --- | --- | --- | --- |
| 1 | Bias due to confounding | Serious | Moderate | Moderate | Serious |
| 2 | Bias in selection of participants into the study | Low | Low | Moderate | Low |
| 3 | Bias in classification of interventions | Low | Low | Low | Low |
| 4 | Bias due to deviations from intended interventions | Low | Low | Low | Low |
| 5 | Bias due to missing data | Serious | Moderate | Serious | Low |
| 6 | Bias in measurement of outcomes | Moderate | Serious | Low | Low |
| 7 | Bias in selection of the reported result | Low | Low | Moderate | Moderate |
| OVERALL RATING | | SERIOUS | MODERATE | MODERATE | MODERATE |

Cross-sectional studies

|  | **AXIS Tool** | **Calo 2020** | **Craft 2000** | **Kubal 2015** |
| --- | --- | --- | --- | --- |
| 1 | Were the aims/objectives of the study clear? | Yes | Yes | Yes |
| 2 | Was the study design appropriate for the stated aim(s)? | Yes | Yes | Yes |
| 3 | Was the sample size justified? | No | No | No |
| 4 | Was the target/reference population clearly defined? | No | No | Yes |
| 5 | Were the risk factor and outcome variables measured correctly using instruments/measurements that had been trialled, piloted or published previously? | Yes | Yes | Yes |
| 6 | Were the methods (including statistical methods) sufficiently described to enable them to be repeated? | Yes | Yes | Yes |
| 7 | Were the results presented for all the analyses described in the methods? | Yes | Yes | Yes |
| 8 | Were the authors' discussions and conclusions justified by the results? | Yes | Yes | Yes |
| 9 | Were the limitations of the study discussed? | Yes | No | No |
| 10 | Was ethical approval or consent of participants attained? | Yes | Yes | No |
| OVERALL RATING | | MODERATE | MODERATE | MODERATE |

Cohort studies

| **Newcastle-Ottawa Tool** | | **Wheeler 2013** |
| --- | --- | --- |
| 1 | Representativeness of the exposed cohort | ★ |
| 2 | Selection of the non-exposed cohort | ★ |
| 3 | Ascertainment of exposure | ★ |
| 4 | Demonstration that outcome of interest was not present at start of study | ★ |
| 5 | Comparability of cohorts on the basis of the design or analysis | ★★ |
| 6 | Assessment of outcome | ★ |
| 7 | Was follow-up long enough for outcomes to occur | ★ |
| 8 | Adequacy of follow up of cohorts | ★ |
| OVERALL RATING | | LOW |

Mixed-methods studies

| **MMAT Tool** | | **Armson 2018** |
| --- | --- | --- |
| 1 | Are there clear research questions? | Yes |
| 2 | Do the collected data allow to address the research questions? | Yes |
| 3 | Is there an adequate rationale for using a mixed methods design to address the research question? | Yes |
| 4 | Are the different components of the study effectively integrated to answer the research question? | Yes |
| 5 | Are the outputs of the integration of qualitative and quantitative components adequately interpreted? | Yes |
| 6 | Are divergences and inconsistencies between quantitative and qualitative results adequately addressed? | ? |
| 7 | Do the different components of the study adhere to the quality criteria of each tradition of the methods involved? | No |
| OVERALL RATING | | MODERATE |

Uncontrolled before-after studies

| **NHLBI-NIH Tool** | | **Bouaud 2001** | **Gilbo 2018** | **Hill 2018** | **Kreizenbeck 2020** | **McWhirter 2007** | **Ottevanger 2004** | **Seroussi 2007** | **Veerbeek 2011** |
| --- | --- | --- | --- | --- | --- | --- | --- | --- | --- |
| 1 | Was the study question or objective clearly stated? | Yes | Yes | Yes | Yes | Yes | Yes | Yes | Yes |
| 2 | Were eligibility/selection criteria for the study population prespecified and clearly described? | Yes | Yes | Yes | No | Yes | Yes | No | Yes |
| 3 | Were the participants in the study representative of those who would be eligible for the test/service/intervention in the general or clinical population of interest? | Yes | Yes | Yes | ? | Yes | Yes | ? | Yes |
| 4 | Were all eligible participants that met the prespecified entry criteria enrolled? | Yes | Yes | Yes | ? | Yes | ? | ? | ? |
| 5 | Was the sample size sufficiently large to provide confidence in the findings? | Yes | ? | Yes | No | Yes | ? | Yes | ? |
| 6 | Was the test/service/intervention clearly described and delivered consistently across the study population? | Yes | Yes | Yes | ? | Yes | Yes | Yes | Yes |
| 7 | Were the outcome measures prespecified, clearly defined, valid, reliable, and assessed consistently across all study participants? | Yes | Yes | Yes | ? | Yes | Yes | Yes | Yes |
| 8 | Were the people assessing the outcomes blinded to the participants' exposures/interventions? | No | No | ? | ? | ? | No | No | ? |
| 9 | Was the loss to follow-up after baseline 20% or less? Were those lost to follow-up accounted for in the analysis? | Yes | Yes | ? | ? | ? | ? | ? | ? |
| 10 | Did the statistical methods examine changes in outcome measures from before to after the intervention? Were statistical tests done that provided p values for the pre-to-post changes? | Yes | No | Yes | No | Yes | Yes | No | Yes |
| 11 | Were outcome measures of interest taken multiple times before the intervention and multiple times after the intervention (i.e., did they use an interrupted time-series design)? | Yes | No | No | No | No | No | No | No |
| 12 | If the intervention was conducted at a group level (e.g., a whole hospital, a community, etc.) did the statistical analysis take into account the use of individual-level data to determine effects at the group level? | No | No | Yes | No | Yes | Yes | No | ? |
| OVERALL RATING | | MODERATE | SERIOUS | LOW | SERIOUS | LOW | MODERATE | SERIOUS | UNCLEAR |
