# Supplementary material for: Characteristics and impact of interventions to support healthcare providers’ compliance with guideline recommendations for breast cancer: a systematic literature review
Source: Implement Sci. 2023 May 22;18:17. doi: 10.1186/s13012-023-01267-2 (PMC10201699; doi:10.1186/s13012-023-01267-2)
Supplement: Supplementary file 4 — Additional file 4. Characteristics and results of the 35 studies included in the review. [file 13012_2023_1267_MOESM4_ESM.docx]

**Additional file 4.** Characteristics and results of the 35 studies included in the review

| **Author(s)/ publication year/ reference** | **Study design** | **Country** | **Setting/ Participants (N)** | **Aim of the study** | **Results** |
| --- | --- | --- | --- | --- | --- |
| **A. *Computerized Clinical Decision Support System*** | | | | | |
| Seroussi et al., 2007 (65) | Uncontrolled before-after study | France | Tenon hospital / 316 medical decisions | To evaluate how the use of OncoDoc2 during MSMs could improve the compliance rate of therapeutic decisions. | Compliance rate significantly increased using OncoDoc2 from 79.2% to 93.4% (p<0.05). |
| Bouaud et al., 2001 (42) | Uncontrolled before-after study | France | Healthcare providers within the Institut Gustave Roussy/ 127 medical decisions | To evaluate the impact of using OncoDoc upon physicians’ prescribing behavior. | Compliance rate significantly increased using OncoDoc from 61% to 85% (p<0.05). |
| Bouaud et al., 2020 (39) | Case study | France | Physicians participating in multidisciplinary boards or tumor boards referring to breast/ 138 patients | To describe the DESIREE project and report preliminary results of the clinical evaluation of its application in three cancer centers (Spain and France). | Compliance rates were very high both without (99.3%) and with (97.1%) the use of the system. |
| Bouaud et al., 2015 (43) | Case study | France | Multidisciplinary meeting physicians in three hospitals/ 394 medical decisions | To study the attitude and reactions from physicians towards the advice of OncoDoc2. | Compliance rate overall was 86.8%. |
| Bouaud et al., 2014 (38) | Case study | France | Six hospitals in the eastern area of Paris/ 557 medical decisions | To evaluate how the way OncoDoc2 is used have an impact on physicians’ compliance with clinical practice guidelines. | Compliance rate overall was 87.8%. Compliance rates were significantly different according to the use of the system: 94.2% among physicians who used it correctly, 80.0% among those who used it incorrectly, and 90.2% among those who didn’t use it (p<0.05). |
| Eccher et al., 2014 (48) | Case study | Italy | Medical Oncology Unit (MOU) of the S. Chiara Hospital of Trento/ 61 medical decisions | To describe and evaluate the performance of OncoCure CDSS. | Compliance rate with the system recommendations was 85.2% |
| Seroussi, Laouenan, et al., 2013 (66); Seroussi et al., 2012 (67) | Case study | France | Tenon hospital/ 1624 medical decisions | To evaluate the compliance rate of BC therapeutic decisions with CancerEst CPGs when OncoDoc2 was used routinely, and to determine patient factors associated with non-compliance. | Compliance rate overall was 91.7%. Factors independently associated with non-compliance included: age > 80 or < 35, microinvasive tumor, presence of microinvasion associated with a unique invasive tumor, prior neoadjuvant treatment, no prior axillary surgery, and HR+/HER+ score. |
| Seroussi, Soulet, et al., 2013 (68) | Case study | France | Six hospitals located within Paris and surrounding areas/ 825 medical decisions | To evaluate the compliance rate of multidisciplinary tumor boards BC therapeutic decisions with CancerEst CPGs when OncoDoc2 was used. | Compliance rate in the intervention arm (routine use of OncoDoc2) was 88% compared to 75% in the control arm (system not available). |
| Bouaud & Seroussi, 2011 (41) | Case study | France | Tenon hospital/ 127 medical decisions | To describe reasons that lead clinicians not to comply with OncoDoc2 recommendations. | Compliance rate while using OncoDoc2 was 91%.  Non-CPG compliance was observed in cases not covered by CPGs (35%); or in overestimation or underestimation of risk when patient data corresponded to threshold values of decision parameters (39%). |
| Bouaud & Seroussi, 2002 (40) | Case study | France | Healthcare providers within the Institut Gustave Roussy/ 127 medical decisions | To adapt OncoDoc to be used in a different site that didn’t take part in the system development; and to evaluate the impact of site-specific recommendations upon physician compliance with the system. | Compliance rate with OncoDoc recommendations was 85%.  No significant differences of compliance observed when site-specific recommendations were (85%) or were not (86%) available. |
| Seroussi et al., 2001 (64) | Case study | France | Pitié-Salpetriere Hospital/ 746 medical decisions | To describe the development and testing of a computerized clinical decision support system (OncoDoc). | Theoretical agreement with OncoDoc recommendations was 96.6%.  Compliance rate (frequency with which the physician followed the recommendation) was 64.3%. |
| ***B. Provider educational intervention*** | | | | | |
| Gorin et al., 2006 (50) | Randomized controlled trial | United States | Physicians with community-based practice from northern Manhattan and the South Bronx/ 168 physicians | To assess the efficacy of academic detailing in increasing recommendations for BC screening in community-based primary care physicians. | Physicians assigned to the intervention were more likely to recommend mammograms (OR 1.85, 95% CI 1.25 – 2.74) and CBEs (OR 2.13, 95% CI 1.31 – 3.46) to women 40 years or older than comparison physicians. |
| Lane et al., 1991 (58) | Not-randomized controlled clinical trial | United States | Primary care practices/ 667 physicians | To examine the feasibility and impact of an intervention to increase BC screening practices in a predominantly fee-for-service practice community in New York. | Compliance rate with mammography referrals of asymptomatic women aged 50 to 75 years significantly improved (p<0.05) in the intervention group (47.9 to 58.2%) but not in the control group (71.4 to 71.4%). |
| Ray-Coquard et al., 2002 (63) | Controlled before-after study | France | Seven hospitals in France/ 994 patients | To evaluate the impact of the CPGs and the implementation strategy in the cancer network; to assess the impact of CPGs on the diagnostic and treatment practice and for breast and colon cancer | Compliance rate with BC guidelines overall significantly improved (p<0.05) in the intervention group (12 to 36%) but not in the control group (6 to 7%). |
| Lane et al., 2001 (57) | Controlled before-after study | United States | Primary care physicians practicing in four suburban/semirural towns on Long Island, New York/ 282 physicians | To test the effect of a CME intervention directed at improving BC screening practices | Compliance with mammography referrals improved among 21% of the intervention and 6% of the control physicians. Compliance with CBE improved among 8% of the intervention and none of the control physicians. Improvement was defined as compliance rate reaching 90-100%. Both comparisons were non-statistically different (p>0.05). |
| Kreizenbeck et al., 2020 (55) | Uncontrolled before-after study | United States | Providers and patients at regional community clinics/ 626 patients | To measure and raise ASCO 2012 recommendation adherence against serum tumor marker tests and advanced imaging for BC survivors who are asymptomatic for recurrence. | Compliance rate significantly improved after the intervention from 77% to 84% (p<0.05). |
| McWhirter et al., 2007 (59) | Uncontrolled before-after study | Canada | Toronto-Sunnybrook Regional Cancer Centre/ 134 patients | To implement and assess an educational intervention to encourage staging guideline utilization in a cohort of early BC patients. | Compliance rates significantly improved after the intervention only for patients in stage I (decrease in chest X-rays from 74% to 37%; bone scans from 57% to 23% and abdominal ultrasounds from 57% to 14%). |
| Calo et al., 2020 (44) | Cross sectional study | United States | Clinicians who completed the course and later responded to the implementation assessment survey/ 96 clinicians | To evaluate the real-world implementation of Strength After Breas Cancer (SABC) in outpatient rehabilitation clinics. | 76% of respondents implemented SABC in outpatient rehabilitation clinics; among those, 93% were still delivering the program two years after receiving the educational intervention. On average, the program was delivered to 13 patients (range 1–60) per clinic. |
| ***C. Audit and feedback interventions*** | | | | | |
| Veerbeek et al., 2011 (69) | Uncontrolled before-after study | The Netherlands | Nine hospitals in the Comprehensive Cancer Centre West area/ 4,364 patients | To investigate the extent to which the clinical audit contributed to the improvement of BC care. | All the nine quality indicators significantly improved (p<0.05) after the intervention with four indicators meeting the norm scores. |
| Craft et al., 2000 (47) | Cross-sectional study | Australia | BC treatment facilities and medical practices/ 221 patients | To improve BC management by facilitating implementation of treatment guidelines. | Compliance rates exceeded 90% for all four indicators (98, 96, 92 and 100%). |
| ***D. Multifaceted interventions*** | | | | | |
| Aspy et al., 2008 (37) | Randomized controlled trial/ | United States | Practices in the Oklahoma Physicians Resources Research Network/ 16 physicians | To apply the best practices research methodology, in combination with a multicomponent implementation intervention to the problem of BC screening with the goal of improving mammography rates. | Compliance rate with documented mammograms improved significantly (p<0.05) in the intervention practices (40 to 52%) compared to the control practices (40 to 35%). In comparison with the control group, improvements were observed in the intervention group both in the proportion of women offered a mammogram (38% vs 53%) and the proportion of women with a recorded mammogram (35% vs 52%). |
| Michielutte et al., 2005 (60) | Randomized controlled trial | United States | Forty-three primary-care practices in central and western North Carolina/ 1,947 patients | To present the results of a practice-based intervention to increase screening mammography among women aged 65 and older who receive medical care in the private sector. | No overall intervention effect observed. Interaction tests indicated a significant program effect for women who were 80 or older, had less than 9 years of education, were black, or had no private insurance to supplement Medicare. |
| Hillman et al., 1998 (54) | Randomized controlled trial | United States | Medicaid health maintenance organization/ 52 primary care centres | To evaluate the impact of feedback and financial incentives on physician compliance with cancer screening guidelines for women aged 50 and older in a Medicaid HMO. | Compliance rate with breast exam increased over time in both the intervention (23.0 to 47.1%) and the control (14.8 to 33.8%) groups. Significant time effect was observed (p<0.05) but no significant between-group effect. |
| Grady et al., 1997 (51) | Randomized controlled trial | United States | Primary care physicians in 61 small community practices/ 95 physicians | To assess the efficacy of behavioral techniques for increasing mammography referrals by primary care physicians in small community practices. | Compliance rates with mammography in both experimental conditions increased over the year while remaining static in the control condition. Annual compliance rates were significantly different (p<0.05) after the intervention between “cue” (62.8%) and control (49.0%) groups. |
| Gilbo et al., 2018 (49) | Uncontrolled before-after study | United States | Multicenter radiation oncology department/1,727 patients | To evaluate the effects of clinical directives that serve as default treatment decisions and prospective contouring rounds on the implementation of HF in a large, multicenter radiation oncology department | Compliance rates increased in the two groups:  In group one (compliance with ASTRO guidelines; n = 685), patients receiving HF increased from 49 to 80%.  In group two (compliance with implemented clinical directives; n = 1042), patients receiving HF increased from 47 to 73%. |
| Hill et al. 2018 (53) | Uncontrolled before-after study | United States | Health facilities part of the Gundersen Medical Foundation, including 30 regional clinics and 5 rural hospitals/ 92 patients | To evaluate an intervention to promote compliance with new National Comprehensive Cancer Network guidelines from routine testing to omission of ordering CBC and LFTs in patients with early BC. | Compliance rates didn’t change significantly after the intervention either for routine CBC (85% to 87%) or LFTs (88 to 92%) ordering. |
| Ottevanger et al., 2004 (62) | Uncontrolled before-after study | The Netherlands | 10 hospitals part of the Comprehensive Cancer Centre East of The Netherlands/ 478 patients | To assess the effect of the audit and feedback, and educational activities on guidelines adherence for premenopausal women with node-positive BC. | Compliance rates significantly improved (p<0.05) for four guideline adherence indicators: patients with ≥10 resected axillary lymph nodes (65 to 81%); reported estrogen receptor status, (85 to 97%); reported progesterone receptor status (83% to 97%); and Dose Intensity of chemotherapy ≥ 85% (75 to 94%). |
| Coleman et al., 2003 (46) | Controlled before-after study | United States | providers in primary care clinics located in 26 of the 27 counties in the Arkansas Delta/ 224 providers | To test a multimethod intervention targeting rural healthcare providers in their clinics to increase BC screening among rural minorities. | Providers in both intervention and control groups improved in demonstration of BC screening practice after the intervention (p<0.05). However, no difference between groups was observed. Compliance rate with mammography in women older than 50 increased in the intervention group. |
| Armson et al., 2018 (36) | Mixed methods study | Canada | Primary care providers members of the Practice-Based Small Group Learning Program/ 70 physicians | To explore which implementation tools (iTools) clinicians used and how often; whether tool use was associated with practice changes; and to identify mediators for practice change(s) related to BC screening. | 92% of participants used tools for clinicians and 62% also used tools for patients. Practice change(s) with iTools use were reported by 77% of participants. As more iTools were used, more practice changes were observed. |
| ***E. Other types of interventions*** | | | | | |
| Chambers et al., 1989 (45) | Randomized controlled clinical trial | United States | Family practice center of the Department of Family Medicine at Thomas Jefferson University/ 1,262 patients | This study investigates the influence of a microcomputer tickler system on the ordering of mammograms | Compliance rate with mammography guidelines was significantly higher in the intervention (27%) compared to the control (21%) group at the study’s completion (p<0.05). Experimental group were more likely to have a mammogram ordered during the study period (19% compared with 12%, P = .001) and more likely to be in compliance with mammography guidelines at the study's completion (27% compared with 21%, P = .011). |
| Wheeler et al., 2013 (70) | Prospective cohort study | United States | Women aged 18 to 64 diagnosed with stage 0, I, II, or unstaged BC from 2003 to 2007/ 840 patients | To explore whether CCNC enrollment was associated with guideline-concordant follow-up care among BC survivors | Increasing months of CCNC enrollment was significantly positively associated with receipt of follow-up mammogram but not with physical examinations/history-taking visits. |
| Kubal et al., 2015 (56) | Cross-sectional study | United States | Providers from the Moffitt Cancer Center/ 66 providers | To report baseline findings at 4 Moffitt Oncology Network sites and discuss how the measurement and feedback system could improve clinical quality and decrease costs in the care of patients with BC. | Compliance rates with MCC pathways varied across sites and between areas, ranging from 11 to 83%. At all 4 sites, 50% or more providers ordered unnecessary tests or procedures as they went through the vignettes. High variability in compliance across centers was observed. |
| Munce et al., 2013 (61) | Case study | Canada | Knowledge Translations Canada Summer Institute/ number of participants not reported | To describe the process of developing an integrated knowledge translation strategy that could be used by guideline developers to improve the uptake of their new clinical practice guidelines on BC screening. | The entire KTA framework was used, but the step involving assessing barriers to knowledge use was judged to be particularly relevant in anticipating implementation challenges and connecting them with appropriate strategies. |
| Groot et al., 2009 (52) | Case study | The Netherlands | Dutch Comprehensive Cancer Centre South and New South Wales BC Institute/ number of participants not reported | To describe a computational method for critiquing clinical actions performed by physicians. | Case study (1): Some deviation was found between the guideline and each of the seven prototypical patient cases. Some differences could be explained by looking at the new revision of the guideline.  Case study (2): The combination of actions prescribed for the case was non-compliant. |

* Detailed description of risk of bias available in Additional File 3

ASCO = American Society of Clinical Oncology; ASTRO = American Society for Radiation Oncology; BC = Breast cancer; CBC = Complete blood cell count; CBE = Clinical breast examination; CCNC = Community Care of North Carolina; CCOPGI = Cancer Care Ontario Practice Guidelines Initiative; CDSS = Clinical decision support system; CME = Continuing medical education; CPGs = Clinical practice guidelines; DESIREE = Decision Support and Information Management System for Breast Cancer; EHR = electronic Health records; HER2 = Human epidermal growth factor receptor 2; HF = Hypofractionation; HR = Hormone receptor; KTA = Knowledge to Action; LFTs = Liver function tests; MCC = Moffitt Cancer Center; MSMs = Multidisciplinary staff meetings; SABC = Strength After Breast Cancer.
