# Supplementary material for: Characteristics and impact of interventions to support healthcare providers’ compliance with guideline recommendations for breast cancer: a systematic literature review
Source: Implement Sci. 2023 May 22;18:17. doi: 10.1186/s13012-023-01267-2 (PMC10201699; doi:10.1186/s13012-023-01267-2)
Supplement: Supplementary file 5 — Additional file 5. Evidence Profiles. [file 13012_2023_1267_MOESM5_ESM.docx]

**Additional File 5. Evidence Profiles**

**5.1. Provider education interventions to support compliance with to breast cancer clinical practice guidelines compared to no intervention for the management of breast cancer**

| **Certainty assessment** | | | | | | | **Impact** | **Certainty** |
| --- | --- | --- | --- | --- | --- | --- | --- | --- |
| **№ of studies** | **Study design** | **Risk of bias** | **Inconsistency** | **Indirectness** | **Imprecision** | **Other considerations** |  |  |
| Compliance rate (evidence from experimental studies) | | | | | | | | |
| 1^1^ | randomised trials | very serious^a^ | not serious | not serious | not serious | none | • Gorin 2006: the intervention improved the recommendation of mammography (OR 1.85, 95% CI 1.25 – 2.74) and clinical breast examination (OR 2.13, 95% CI 1.31 – 3.46) in female patients aged 40 and over. | ⨁⨁◯◯ Low |
| compliance rate (evidence from observational studies) | | | | | | | | |
| 3^2,3,4^ | observational studies | serious^b^ | not serious | not serious | not serious | none | • Ray-Coquard 2002: improved compliance to diagnostic and treatment CPG recommendations in the intervention group (from 12% before the intervention to 36% post-intervention; P<0.001), whereas no significant improvements were observed in the control group.  • Lane 2001: significant (p<0.05) improvements in providers’ knowledge, attitudes, and self-efficacy towards the new CPG recommendations in the intervention group  • Lane 1991: Significant improvement in the number of reported mammography referrals of asymptomatic women aged 50 to 75 years in the intervention group but not in the control group | ⨁◯◯◯ Very Low |

**Explanations**

a. The only RCT identified (Gorin et al., 2006) presented a high risk of bias due to concerns due to the generation of random sequence, allocation concealment, and blinding.

b. The three studies identified (Lane 1991; Lane 2001; Ray-Coquard 2002) are controlled before-after studies with a moderate risk of bias due to: serious risk of confounding (Ray-Coquard 2002), missing data (Lane 2001), and outcome measurement (Lane 1991).

**References**

1.Gorin, S. S., Ashford, A. R., Lantigua, R., Hossain, A., Desai, M., Troxel, A., Gemson, D.. Effectiveness of academic detailing on breast cancer screening among primary care physicians in an underserved community. J Am Board Fam Med; Mar-Apr 2006.

2.Ray-Coquard, I., Philip, T., de Laroche, G., Froger, X., Suchaud, J. P., Voloch, A., Mathieu-Daudé, H., Fervers, B., Farsi, F., Browman, G. P., Chauvin, F.. A controlled &quot;before-after&quot; study: impact of a clinical guidelines programme and regional cancer network organization on medical practice. Br J Cancer; Feb 1 2002.

3.Lane, D. S., Polednak, A. P., Burg, M. A.. Effect of continuing medical education and cost reduction on physician compliance with mammography screening guidelines. J Fam Pract; Oct 1991.

4.Lane, D. S., Messina, C. R., Grimson, R.. An educational approach to improving physician breast cancer screening practices and counseling skills. Patient Educ Couns; Jun 2001.

**5.2. Provider reminders to support compliance with breast cancer clinical practice guidelines compared to no intervention for the management of breast cancer**

| **Certainty assessment** | | | | | | | **Impact** | **Certainty** |
| --- | --- | --- | --- | --- | --- | --- | --- | --- |
| **№ of studies** | **Study design** | **Risk of bias** | **Inconsistency** | **Indirectness** | **Imprecision** | **Other considerations** |  |  |
| Compliance rate | | | | | | | | |
| 1^1^ | randomised trial | not serious | not serious | serious^a^ | not serious | none | A microcomputer-generated reminder system for ordering mammograms improved compliance with mammography guidelines: 27% (170/639) in the intervention vs 21% (128/623) in the control group (p = 0.011) after six months follow-up. OR= 1.40 (95%CI=1.01 to 1.82) | ⨁⨁⨁◯ Moderate |

**Explanations**

a. The available evidence is restricted to only one type of reminder system and does not inform other types of reminders.

**References**

1.Chambers, C. V., Balaban, D. J., Carlson, B. L., Ungemack, J. A., Grasberger, D. M.. Microcomputer-generated reminders. Improving the compliance of primary care physicians with mammography screening guidelines. J Fam Pract; Sep 1989.

**5.3. Multifaceted interventions to support compliance with breast cancer clinical practice guidelines compared to no intervention for management of breast cancer**

| **Certainty assessment** | | | | | | | **Impact** | **Certainty** |
| --- | --- | --- | --- | --- | --- | --- | --- | --- |
| **№ of studies** | **Study design** | **Risk of bias** | **Inconsistency** | **Indirectness** | **Imprecision** | **Other considerations** |  |  |
| Compliance rate (evidence from experimental studies) | | | | | | | | |
| 4^1,2,3,4^ | randomised trials | serious^a^ | serious^b^ | not serious | not serious | none | • Aspy 2008: In comparison with usual care, a multifaceted intervention (including audit and feedback; provider education; information technology support) increased the proportion of women offered a mammogram (38% vs 53%), and the proportion of women with a recorded mammogram (35% vs 52%)  • Michielutte 2005: A multifaceted intervention (comprising provider education and patient education through pamphlets), did not improve compliance with screening mammography guidelines in the overall sample, but produced significant improvements in specific vulnerable subgroups (elderly, lower educational attainment, black ethnicity and with no private insurance)  • Hillman 1998: A multifaceted intervention (audit and feedback plus financial incentives) significantly increased the screening rates both in the intervention and control groups, with no statistically significant differences observed between groups. Compliance rates for mammography recommendation in the intervention and control group were 40.9 and 34.4 at baseline; and 63.6 and 58.6.at post intervention. For breast exam recommendation, the compliance rate in the intervention and control group were 23 and 14.8 at baseline; and 47.1 and 33.8 at post-intervention.  • Grady 1997: A multifaceted intervention (provider education, cue enhancement plus feedback, and token rewards) observed that mammography compliance rates significantly improved (p<0.05) in the intervention (62.8%) in comparison with the control (49.0%) group. | ⨁⨁◯◯ Low |
| Compliance rate (evidence from observational evidence) | | | | | | | | |
| 1^5^ | observational studies | veryserious^c^ | not serious | not serious | not serious | none | • Coleman 2003: A multifaceted intervention (including audit and feedback, patient and professional education) improved the demonstration of breast cancer screening, with significantly more women older than 50 receiving mammograms in the intervention than in the comparison group. | ⨁◯◯◯ Very low |

**Explanations**

a. Of the four RCTs identified, Hilman 1998 presented a low risk of bias, Aspy 2008 moderate risk of bias (due to lack of blinding), and Grady 1997 and Michielutte 2005 presented an unclear risk of bias (incomplete reporting)

b. Effects range from clear benefit (Aspy 2008; Grady 1997) to no benefit (Hillman 1998)

c. A controlled before-after study (Coleman 2003) presented a serious risk of bias (confounding bias and bias arising from missing data)

**References**

1.Michielutte, R., Sharp, P. C., Foley, K. L., Cunningham, L. E., Spangler, J. G., Paskett, E. D., Case, L. D.. Intervention to increase screening mammography among women 65 and older. Health Education Research; Apr 2005.

2.Hillman, A. L., Ripley, K., Goldfarb, N., Nuamah, I., Weiner, J., Lusk, E.. Physician financial incentives and feedback: failure to increase cancer screening in Medicaid managed care. Am J Public Health; Nov 1998.

3.Grady, K. E., Lemkau, J. P., Lee, N. R., Caddell, C.. Enhancing mammography referral in primary care. Preventive Medicine; Nov-Dec 1997.

4.Aspy, Cheryl B., Enright, Margaret, Halstead, LaWanna, Mold, James W.. Improving mammography screening using best practices and practice enhancement assistants: An Oklahoma Physicians Resource/Research Network (OKPRN) study. Journal of the American Board of Family Medicine; Jul-Aug 2008.

5.Coleman, E. A., Lord, J., Heard, J., Coon, S., Cantrell, M., Mohrmann, C., O'Sullivan, P.. The Delta project: increasing breast cancer screening among rural minority and older women by targeting rural healthcare providers. Oncol Nurs Forum; Jul-Aug 2003.
